# Bioprospecting Cultivated Tropical Green Algae, *Caulerpa racemosa* (Forsskal) J. Agardh: A Perspective on Nutritional Properties, Antioxidative Capacity and Anti-Diabetic Potential

**DOI:** 10.3390/foods9091313

**Published:** 2020-09-18

**Authors:** Abdul Qudus B Aroyehun, Shariza Abdul Razak, Kishneth Palaniveloo, Thilahgavani Nagappan, Nur Suraiza Nabila Rahmah, Gan Wee Jin, Dinesh Kumar Chellappan, Jestin Chellian, Anil Philip Kunnath

**Affiliations:** 1 Nutrition and Dietetics Program, School of Health Sciences, Health Campus, Universiti Sains Malaysia, Kubang Kerian 16150, Kelantan, Malaysia; bqaroyehun@student.usm.my; 2 Institute of Ocean and Earth Sciences, Institute for Advanced Studies Building, University of Malaya, Wilayah Persekutuan, Kuala Lumpur 50603, Malaysia; 3 Faculty of Marine and Environmental Sciences, Universiti Malaysia Terengganu, Kuala Terengganu 21030, Terengganu, Malaysia; 4 Institute of Marine Biotechnology, Universiti Malaysia Terengganu, Kuala Terengganu 21030, Terengganu, Malaysia; 5 School of Pharmacy, International Medical University, Bukit Jalil, Kuala Lumpur 57000, Malaysia; suraizanabila@gmail.com (N.S.N.R.); eugenegan77@gmail.com (G.W.J.); 6 Department of Life Sciences, International Medical University, Bukit Jalil, Kuala Lumpur 57000, Malaysia; dinesh_kumar@imu.edu.my (D.K.C.); jestin_chellian@imu.edu.my (J.C.); 7 Division of Applied Biomedical Science and Biotechnology, School of Health Sciences, International Medical University, Bukit Jalil, Kuala Lumpur 57000, Malaysia; anilphilip_kunnath@imu.edu.my

**Keywords:** seaweed, *Caulerpa racemosa*, chemical composition, nutrition, antioxidant, anti-diabetic

## Abstract

*Caulerpa racemosa* (Forsskal) J. Agardh is a green seaweed used as food and folk medicine since ancient times in the Indo-Pacific region, particularly in southeast Asia. In this study, the proximate nutrient composition, phytochemical, anti-oxidant and anti-diabetic properties of sea grape *C. racemosa* collected from culture fishponds in Johor, Malaysia were analysed. The contents (dry weight basis) of carbohydrate, crude protein, crude lipids, ash and caloric value obtained were 33.42 ± 1.34%, 20.27 ± 0.14%, 4.20 ± 0.32%, 28.25 ± 0.27% and 2544.67 ± 7.04 cal g^−1^, respectively. The amino acid score (AAs) and biological protein value (213.43 mg g^−1^) indicated that *C. racemosa* presented a better protein quality. The most abundant fatty acids were C16:0 (palmitic acid: 63.27%), followed by C18:1 (oleic acid: 5.80%), and C18:2 ῳ6 (linoleic acid: 5.33%). The analysis of the ash content indicated that essential minerals and trace elements, such as Ca, Fe, and Mn, were present in the seaweed. The total phenolic content (TPC) and total flavonoid content (TFC) observed in the ethyl acetate extract were 17.88 ± 0.78 mg GAE g^−1^ and 59.43 ± 2.45 mg QE g^−1^, respectively. The ethyl acetate extract of *C. racemosa* demonstrated notable anti-diabetic activity in diabetic induced rats. The low (100 mg kg^−1^) and high (200 mg kg^−1^) doses of cultivated *C. racemosa* extract exhibited a significant decrease (*p* < 0.05) in blood glucose levels while preventing weight loss, reducing plasma AST, ALT levels as a sign of hepatoprotective effect and recording albumin levels similar to positive control in diabetic induced rats. The results support the usefulness of cultivated *C. racemosa* as a potential functional food.

## 1. Introduction

Marine macroalgae, commonly known as seaweed, is a phylogenetically diverse group important to maintain oceanic balance [1]. Seaweed meadow provides shelter and habitat to different marine organisms for all or part of their life-cycle [2]. As primary producers, seaweeds play an important ecological role of providing nutrients and energy to marine organisms, both directly or indirectly [3]. Seaweeds are a potential source of macro and micronutrients, containing high-quality proteins, soluble dietary fiber, vitamin constituents, minerals, phytochemicals and fatty acids which offer protection against numerous neurodegenerative pathologies [4]. In recent years, technological and research advancements have promoted exploration of seaweed as functional food, energy, pharmaceuticals and medicine [5,6]. Metabolomics application has provided useful insights to discovery of novel bioactive compounds [7].

Approximately 12,272 algae species are classified as Chlorophyta (green algae), the Phaeophyta (brown algae) and Rhodophyta (red algae) based on their pigmentation. Seaweed can be cultured in aqualture systems in enclosed areas or directly in the sea. Some aqualculturist co-culture seaweed with commercially viable aquatic organisms such as abalone farming in integrated multi-trophic aquaculture systems for proper management and sustainability of their nutritional characteristics and composition [8]. The green algae with 4548 species is the least exploited despite being the group with the closest relationship to higher plants [9]. In Malaysia, a total eight species of Caulerpa has been reported; *C. lentillifera*, *C. peltata*, *C. racemosa*, *C. scalpelliformis*, *C. serrulata*, *C. sertulariodes*, *C. taxifolia* and *C. verticillata* [10]. The species *C. racemosa*, which is commonly referred to as sea grapes is a local delicacy, is traditionally consumed as salad or vegetable among coastal communities in southeast Asian countries such as Indonesia, the Philippines and Malaysia (Sabah) [11]. *Caulerpa racemosa* is known for its high nutritional value, including rich polyunsaturated fatty acids (PUFA), essential amino acids, minerals, dietary fibers, vitamins and natural bioactive compounds [12,13]. The presence of natural bioactive compounds in *C. racemosa* have contributed to its antioxidant, anticoagulant, antimutagenic, antibacterial and anticancer activities [13,14,15,16].

*Caulerpa racemosa* (Forsskal) J. Agardh stock is fast dwindling due to extensive harvesting for use as food and feed [17]. As an important functional food, *C. racemosa* is widely cultured, under modified conditions. Even natural populations tend do have varying properties due to changes in environmental conditions such as sedimentation, salinity, temperature and pollution [18]. During our regular sampling trip to Merambong shoals of Johor waters, we came across healthily growing *C. racemosa* in earthen fish culture ponds at Tanjung Kupang. Under conditions where the growth of seaweed depends on nutrients from fish feed and discharge, the nutritional and biochemical properties of the earthern pond *C. racemosa* was expected to vary compared to natural specimens in the wild. Since literature does not report characteristics of *C. racemosa* under similar growth conditions, this research will evaluate the nutritional value, phytochemical properties and bioactivity of *C. racemosa* extract as an anti-oxidative and anti-diabetic agent in order to assess its potential as functional food. This is perhaps the only research paper that has delved into nutritional properties, antioxidant capacity and anti-diabetic potential in one report.

## 2. Materials and Methods

### 2.1. Collection, Identification and Processing of Algal Material

*Caulerpa racemosa* growing in three earthen ponds (*n* = 3) at Tanjung Kupang, Johor, approximately 1 kg wet weight (kg *w/w*) each, was hand-picked and treated separately with assistance from volunteers of the *Kelab Alami Mukim Tanjung Kupang* in January 2018. Samples identification was confirmed based on morphological identification and description from Verlaque et al. [19], Belleza and Liao [20] and Zubia et al. [21]. A herbarium voucher (UMTP1901) was stored at the Faculty of Marine and Environmental Sciences herbarium of Universiti Malaysia Terengganu. The seaweed samples were cleaned in freshwater to remove sediment and then deep freezed at (−20 °C) for 48 h prior to freeze-drying (ModulyoD, Thermo Electron Corporation, Waltham, MA, USA) for another 48 h. Freeze-dried algae samples were ground in a mechanical grinder (IKA, A 11 Basic, Berlin, Germany), to obtain homogeneous powder (500 μm), kept at −40 °C) until further use.

### 2.2. Proximate Analysis

Moisture content was determined gravimetrically by measuring sample (3 g) weight loss by drying in an oven (Memmert UFP 600, Buechenbach, Germany) at 105 °C) until a constant weight was obtained (AOAC 934.01). Ash content was calculated after incineration in a muffle furnace (Barnstead Thermolyne) for 18 hrs at 550 °C) [22]. Total protein content of samples was quantified according to the Kjeldahl method (N ×5) using a Foss Kjeltec system (FOSS, Hilleroed, Denmark) [23]. Total lipids were obtained by reflux soxhlet extraction with chloroform: methanol 2:1 (*v/v*) for 4 h. The carbohydrate contents were calculated using the formula:

Carbohydrates = [100% − (% protein +% lipid +% ash %+ moisture)]

The gross calorific content was estimated using the Isoperibol oxygen bomb calorimeter (IKA Calorimeter System C 2000 basic, Staufen, Germany) standardized with benzoic acids. One gram (g) of freeze-dried algae pellets was combusted in oxygen at 200 bar (2900 PSI) with a core maximum temperature of 1000 °C (1800 F). Total calories were calculated on an ash-free basis [24]. The results were expressed in dry weight (DW) basis and all measurements were performed in triplicate. Vitamin B2 content was quantified using an Agilent 1260 Series HPLC system (Agilent, USA) at at 270 nm using a Onyx Monolithic C18 column (100 × 4.6 mm i.d) [25], analysed over a gradient mobile phase 12% to 100% acetonitrile (solvent B) in HPLC grade water (solvent A) with a flow of 1 mL min^−1^ for 12 min and compared to standards.

### 2.3. Fatty Acid Determination

Lipid was extracted from *C. racemosa* using chloroform: methanol (2:1, *v/v*). Fatty acid was eluted from the crude lipid by running through a silica gel column chromatography using a mobile phase of Hexane:Ethyl acetate (9:1). The fatty acids (FAs) were converted to methyl esters by transmethylation using sodium methoxide solution. Precisely 100 mg of concentrated fatty acid extract was converted to FAME through methylation by adding 2.7 mL of Hex and 0.3 mL of 2 M sodium methoxide solution in a methylation vial. The vial is then subjected to constant mixing using a magnetic stirrer at room temperature for 3 h. The chloroform-methanol (1:1) mixture was added into the FAMEs obtained to stop the reaction. The mixture was dried using a rotary evaporator and a yellowish oil is obtained. The FAMEs were analyzed using a Shimadzu QP-2010 gas chromatograph (GC) equipped with a silica BPX70 capillary column (60 m, with a film thickness of 0.25 μm) coupled with mass chromatography (MS) detector using helium as carrier gas. The run method was executed through a temperature gradient between 150 to 240 °C over 60 min. A five point calibration curve was prepared, and the sample was analysed in triplicate for accuracy. Identification and quantification of FAMEs were accomplished by comparing the retention times of the peaks with those of pure FAME standard (Sigma-Aldrich, St. Louis, MO, USA) analyzed under the same conditions. Concentration of FAME was calculated based on GC area of a peak compared to standard used and expressed as a percentage of individual FAs in the lipid fraction [13].

### 2.4. Amino Acid Analysis

Accurately, 0.1 g of powdered *C. racemosa* was hydrolysed with 1 mL of 6 N hydrochloric acid (HCl) in a water bath at 100 °C, followed by addition of 1 mL 0.1 N HCl: ethanol (EtOH) mixture (1:1, v:v) in an ice-water bath. Aliquots (25 μL) of hydrolysates were mixed with 25 μL of 10 N sodium hydroxide (NaOH), followed by the addition of 1 mL of derivatization reagent (solution mixture of ortho-phthalaldehyde (OPA) 1.25 × 10^−2^ M and N-acetylcysteine (NAC) 2.5 × 10^−2^ M buffered with 1 M boric acid at pH 9.5) [26]. 20 μL of the 0.45 μm filtered derivatized amino acids was analysed using a High Performance Liquid Chromatography (HPLC) at 335 nm ultra violet range and a C18 column (i.d., 4.6 × 180 mm, Agilent Technologies, Santa Clara, CA, USA) at 40 °C. The mobile phase consisted of 5 mM citric acid in water adjusted to pH 6.5 (mobile phase A) and acetonitrile (mobile phase B) set for the following profile: Mobile phase A: 95% at time 0 min, 70% at 30 min, 50% at 35 min and 95% from 37 min until 45 min). Amino acid standards (Sigma-Aldrich, Missouri, USA ) were similarly analysed for reference and quantification in samples. A three-point calibration curve was prepared, and the sample was analysed in triplicate for accuracy. Identification and quantification of FAMEs were accomplished by comparing the retention times of the peaks with those of pure standards (Sigma-Aldrich, St. Louis, MO, USA). Concentration of amino acids was calculated based on peak area with reference to standard and expressed as a percentage.

### 2.5. Mineral and Heavy Metal Analysis

Accurately, 0.5 g freeze-dried *C. racemosa* was subjected to wet hydrolysis using a high-pressure polytetrafluoroethylene (PTFE) vessel, containing 6 mL of 65% HNO_3_ and 2 mL of 35% H_2_O_2_ and digested in an Anton Paar microwave. After digestion, samples were filtered and diluted with Milli-Q water to a final volume of 50 mL and analysed in a Agilent 7700 series ICP-MS (Agilent, United States of America) for multi-mineral elements. The total concentrations of freeze-dried seaweed minerals were then quantified from calibration curves of their respective standard elements [27].

### 2.6. Antioxidative Properties

#### 2.6.1. Total Phenolic Content (Tpc) and Flavonoid Content (Tfc)

Total phenolic content was determined by using a Folin–Ciocalteau (FC) assay where 1 mL of methanolic extracts stock solution was added to distilled water (9 mL) followed by 1 mL of FC reagent (10 times diluted with distilled water). After 5 min, 10 mL 7% Na_2_CO_3_ was added to the mixture, kept for 30 min, and the absorbance was measured at 750 nm using a UV spectrophotometer. A calibration curve of Gallic acid (25–200 μg mL^−1^) was prepared (R^2^ = 0.999), and the percentage of total phenolics was calculated from the calibration curve of gallic acid and expressed as mg gallic acid equivalent (GAE) g^−1^ dried plant material [28]. Total flavonoid content was determined by using the aluminium chloride colorometric assay where 1 mL extract stock solution was added to distilled water (5 mL), followed by 0.3 mL 5% NaNO_2_. After 5 min, 0.6 mL 10% AlCl_3_ and 2 mL 1 M NaOH was added. The absorbance was measured at 510 nm and percentage of total flavonoids was calculated based on calibration curve of quercetin and expressed as mg quercetin equivalent (QE) g^−1^ dried material [29]. A calibration curve of quercetin (25–250 μg mL^−1^) was prepared (R^2^ = 0.997).

#### 2.6.2. Estimation of Total Antioxidant Activity

Approximately 0.3 g of *C. racemosa* EtOAc extracts were mixed with 3.0 mL solution comprised of 28 mM sodium phosphate, 0.6 M sulfuric acid, and 4 mM ammonium molybdate. This mixture was incubated at 95 °C for 90 min in a water bath. The absorbance of mixture was measured at 695 nm using a UV-Vis 2450 spectrophotometer (Shimadzu, Japan) and total antioxidant activity is expressed as the number of equivalents of ascorbic acid in milligram per gram of extract (mg L-ascorbic acid g^−1^) [30]. The standard curve of ascorbic acid was linear between 50 and 250 μg mL^−1^ (R^2^ = 0.994).

#### 2.6.3. Estimation of Ferric Reducing Antioxidant Power (Frap)

Accurately, 0.5 mL of *C. racemosa* EtOAc extracts was diluted with 1.25 mL of potassium ferricyanide (K_3_Fe(CN)_6_ (10 mg mL^−1^) and 1.25 mL of phosphate buffer (0.2 M, pH = 6.6). The mixture was then incubated at 50 °C for 20 min in a water bath. Subsequently, 1.25 mL of 10% trichloroacetic acid was then added to the mixture and agitated before the mixture was centrifuged at 3000-rpm for 10 min. The residue was discarded while the supernatant (1.0 mL) was further diluted with 0.5 mL of distilled H_2_0 and 0.25 mL of freshly prepared (0.1% w/v) FeCl_3_.6H_2_O solution. After 10 min, the absorbance was measured at 700 nm using a UV-Vis 2450 spectrophotometer (Shimadzu, Japan) [31]. Ascorbic acid was used as the standard. The standard curve of ascorbic acid was linear between 50 and 250 μg mL^−1^ (R^2^ = 0.994).

#### 2.6.4. Estimation of Hydrogen Peroxide (H _2_O _2_)

Carefully, 0.1 mL of *C. racemosa* EtOAc extracts were mixed with 0.4 mL phosphate buffer (50 mM, pH 7.4) and 0.6 mL H_2_O_2_ (50 mM). The mixture was vortexed, and absorbance of the H_2_O_2_ was determined after 10 min at 230 nm. Ascorbic acid was used as a control [31].The standard curve of ascorbic acid was linear between 50 and 250 mL^−1^ (R^2^ = 0.996) The abilities to scavenge the H_2_O_2_ were calculated using the following equation:

H_2_0_2_ scavenging activity = (1 − absorbance of sample/absorbance of sample) × 100

### 2.7. In Vivo Anti-Diabetic Study

As a functional food, initial extraction of *Caulerpa racemosa* used ethanol. Prior to this in vivo study, preliminary screening using colorimetric enzyme inhibitory assays; α-amylase, α-glucosidase were previously done to verify literature claims and possible anti-diabetic potential. This being the reference, the initial in vivo evaluation of anti-diabetic potential using ethanolic extract recorded mortality. Therefore, a subsequent experiment was performed using samples extracted over ethyl acetate. Extraction was done for at least for 24 h in room temperature prior to filtration and concentration under reduced pressure using a rotary evaporator with water bath temperature set at not exceeding 40 °C. Crude extract was kept at room temperature until further use. Ethyl acetate was used as a preferred solvent for extraction as our preliminary study on *C. racemosa* ethyl acetate extract showed the presence of significant amounts of flavonoids and phytophenols.

#### 2.7.1. Experimental Animals

Thirty (30) eight weeks old (150–200 g) male Sprague Dawley (SD) rats were procured from Universiti Putra Malaysia and housed in plastic cages in a well-ventilated room under controlled environment (temperature: 30 ± 2 °C, photoperiod: 12:12 h light/dark cycle) at the International Medical University Malaysia. The rats were provided standard diet and water ad libitum. The animal model assay was approved by the local ethical committee of International Medical University Malaysia (BP I-01-2019(35)).

#### 2.7.2. Experimental Design

Thirty SD rats were divided into five groups of six rats each. The groupings are displayed in Table 1. Group I was the control. Diabetes was induced in Group II to Group V using STZ. Diabetes was induced into overnight (12 h) fasted rats via intraperitoneal injection of streptozotocin (STZ) (65 mg kg^−1^) dissolved in 12 mL of 0.9% saline solution [32]. All groups were induced except the normal group. The diabetic state of the animals was confirmed by checking the blood glucose level of the animals by the tail-vein method. A small amount of blood was obtained from the tail vein of the animals and then was assessed using an Accu-Chek Instant S glucometer (Roche Diabetes Care, Inc., Indianapolis, IN, USA), 10 days after diabetic induction. Animals that showed blood glucose levels higher than 11.1 mmol L^−1^ were considered diabetic and were included in this study. As a standard drug treatment, 180 mg kg^−1^ of metformin was administered to Group V [33]. Group III (low dose) and Group IV (high dose) were experimental rats treated with 100 mg kg^−1^ and 200 mg kg^−1^ of *C. racemosa* extracts, respectively. The doses were determined based on recommended doses for antidiabetic evaluation in mice by Eddouks et al. [34]. The experiment is designed for a 14-day treatment period, and all doses were supplied orally via feeding tube once daily.

#### 2.7.3. Oral Glucose Tolerance Test (OGTT)

An oral glucose load of 3 g kg^−1^ body weight was fed to rats in each of the 5 groups. Blood glucose was measured at 0, 30, 60, 90 and 120 min time intervals after being treated with glucose [35]. Blood samples were obtained by pricking the tip of the rats’ tails with a 26G needle, and glucose levels were assessed with blood glucose test strips on an Accu-Chek Instant S glucometer (Roche Diabetes Care, Inc., Indianapolis, IN, USA).

### 2.8. Statistical Analysis

All the experiments were performed in triplicate analysis and represented as Mean ± SD. Anti-diabetic data were further analyzed using one-way ANOVA via GraphPad Prism 8.2.1.

## 3. Results and Discussion

### 3.1. Proximate Composition of C. racemosa

The average protein and ash contents of analysed *C. racemosa* samples were (20.27 ± 0.14% DW and 28.25% DW), respectively, slightly higher compared to reports for wild *C. racemosa* (17.28 ± 0.63% DW and 26.74 ± 2.17% DW) harvested from Sabah, Malaysia [13]. The values were higher than its tropical to subtropical species collected off the Coast of Naozhou Island, South China Sea with protein and ash composition valued at 11.39 ± 0.32% DW and 7.97 ± 0.46% DW, respectively [36]. Other species of the genus *Caulerpa* recorded varying composition; i.e., *C. lentillifera* (6.6% DW and 48.9% DW) [37] and *C. scalpelliformis* (10.50 ± 0.91 DW and 40.77 ± 2.15% DW) and *C. veravelensis* (7.77 ± 0.59 DW and 33.70 ± 2.73% DW) [14]. High ash content indicates the presence of significant amount of diverse minerals [38], while the protein content in the algae makes it an important source of protein in the marine environment [39]. Protein regulates algal biological processes, and their activities can be characterised by enzymatic catalysis, transport and storage [40]. Seaweeds are highly perishable within days of harvesting, thus proper drying plays an important role in preserving their nutritional status. Moisture content in cultivated *C. racemosa* was 13.85 ± 0.93% in the range similar to a sub-tropical species at 15.37 ± 0.72% [41]. Moisture content of seafoods strongly affects their microbiological and chemical stability, physical properties, and is also used to determine their nutritional composition [42]. Crude lipids content of *C. racemosa* was 4.20 ± 0.32% DW varies greatly when compared to reports from literature. Lipid contents are reported as high as 19.1% DW [43] and can be as low as 2.21 ± 0.05% DW ([13]) and (1.03 ± 0.01% DW) (Hao et al., 2019). Carbohydrate content in the studied *C. racemosa* was calculated at 33.42 ± 1.34% DW. Carbohydrate content in *Caulerpa* has been reported to vary from 3.6–83.2% DW [38]. As an important energy, carbohydrate is vital for metabolic processes [44]. Complete combustion of the *C. racemosa* sample recorded energy values as high as 2544.67 ± 7.04 cal g^−1^ and was higher than most reports of *Caulerpa* species from the Indian coast which ranged from 8.70 ± 0.56–1091 ± 1.74 cal g^−1^[14]. The high protein content quantified could be a contributing factor to the high calorific value of the sample. The *C. racemosa* samples recorded levels of vitamin B2 (riboflavin) at a concentration of 2.03 ± 0.06 mg kg^−1^ DW. The vitamin B2 composition in the samples analysed were similar to previous reports of *C. racemosa* from the west coast of Malaysia [45]. Vitamin B2 is a vital component of the nucleic acid cofactor i.e., FMN (flavin mononucleotide) and FAD (flavin adenine dinucleotide) involved in several cellular functions including electron transport and reactions involving synthesis of niacin and folate [39]. The proximate composition of *C. racemosa* from the collected ponds is summarized in Table 2.

### 3.2. Fatty Acids Composition

Fatty acid compositions of investigated *C. racemosa* are presented in Table 2. The cultured *C. racemosa* contained 80.59 ± 0.10% saturated fatty acids (SFA), followed by 12.24 ± 0.07% monounsaturated fatty acid (MUFA) and 7.17 ± 0.07% polyunsaturated fatty acid (PUFA). Nagappan and Vairappan (2014) [13] also reported a similar profile in the population of *C. racemosa* collected from East Malaysia which contained SFA in the range of 57.83 ± 3.85–66.31 ± 3.21%, MUFA in the range of 12.25 ± 0.88–12.90 ± 1.51%, and PUFA in the range of 29.68 ± 3.72–30.27 ± 3.98%. Similarly, the concentration levels of SFA, MUFA, and PUFA in *C. racemosa var peltata* were recorded at 48%, 25.72%, and 26.33%, respectively [36].

Among the fatty acids, palmitic acid (C16:0) was quantified as the major fatty acid species with 63.27 ± 0.21% followed by oleic acid (C18:1) at 5.80 ± 0.10% and linoleic acid (C18:2 ω6) at 5.33 ± 0.06%. This was in accordance with a previous study where palmitic acid (C16:0), linoleic acid (C18:2), and linolenic acid (C18:3) were the most abundant fatty acids found in the cultivated *C. racemosa* population collected from Australia [17]. The wild population of *C. racemosa* from India on the other hand recorded high amounts of palmitic acid (C16:0), followed by palmitoleic acid (C16:1) and arachidonic acid (C20:4) [14]. Variations in the fatty acid composition of seaweeds can be attributed to changes in environmental conditions including habitat, salinity, light irradiation, seasonality, species and genetic status [46,47]. Recent studies have shown that fatty acid profiles were specific to taxonomic groups [48,49]. The presence of other essential fatty acids such as oleic acid (C18:1ω9), linoleic acid (C18:2ω6-cis), linolenic acid (C18:3ω3), and arachidonic acid (C20:4ω6) obtained in our samples suggests that dietary consumption might be important for good health and normal body development. The ratio of total ω6 fatty acids to total ω3 fatty acids (ω6/ω3) was measured to be (2.91 ± 0.03). This was in agreement with previous data reported by Nagappan and Vairappan (2014) in *C. racemosa* (2.90 ± 0.07–3.25 ± 0.02) [13]. The World Health Organisation (WHO) currently recommends that the ratio (ω6/ω3) should not be higher than 10 in the diet as a whole [14], suggesting that the cultivated *C. racemosa* inviestigated in this study may be used as an alternative functional food to reduce the ω6/ω3 ratio in the daily diet. Generally, seaweeds recording low ω6/ω3 ratio of fatty acids could help to prevent the growth of atherosclerotic plaque and decrease low-density lipoproteins as well as blood cholesterol which may adversely induce heart disease risk [49,50].

### 3.3. Amino Acid Composition

The total content of amino acid quantified in the seaweed collected was 159.69 ± 7.41 mg g^−1^ DW) (15.97% DW). This value was considerably lower than the crude protein levels (Table 1), suggesting that the amount of non-protein nitrogenous material found in this seaweed was significant. This limitation of the micro Kjeldahl method has been debated to overestimate the total protein content due to the presence of non-protein nitrogen constituents such as photosynthetic pigments, nucleic acids, free AA and inorganic nitrogen (nitrate, nitrite and ammonia) [22,51,52,53]. The cultivated *C. racemosa* contained a substantial level of essential amino acids (EEA) such as leucine (2.34 ± 1.16 mg g^−1^ of protein DW), threonine (1.42 ± 0.60 mg g^−1^ of protein DW), valine ( 1.42 ± 0.49 mg g^−1^ of protein DW) and phenylalanine (1.24 ± 0. 43 mg g^−1^ of protein DW). The six essential amino acids (EAAs) quantified appeared in the following order of concentration; leucine > threonine > valine > phenylalanine > isoleucine > lysine, which accounted for 4.80% of the total amino acids in the seaweed. Histidine, methionine, and tryptophan showed no detectable amounts in the EAAs fraction suggesting they were eliminated during acid hydrolysis. Since cystine requirement can be replaced by methionine and similarly between tyrosine and phenylalanine, these amino acids (i.e., methionine with cysteine and tyrosine with phenylalanine) were combined in the present study for calculation of chemical score [54].

Generally, sulfur amino acids are considered as limiting amino acids to their insufficient natural amounts [55], but the seaweed collected recorded lysine as the most limiting amino acid (AA) found in *C. racemosa*. This is in agreement with the data found by Matanjun et al. (2009) [50] which also reported that the most limiting EAA in the green seaweed *Caulerpa* spp. was lysine. However, Bhuiyan et al. [41] observed that methionine was the most limiting AA found in *C. racemosa*. The ratio value of EAA/NEAA was 0.05, and these values indicate that content of EAAs was lower in concentrations than NEAAs. Based on the reference value of 0.6 recommended by the FAO/WHO, the low ratio in *C. racemosa* indicates that the seaweed does not contribute to the required amount of amino acids for the human diet [56]. As for the NEAA profile, glutamic acid (15.49 ± 3.87 mg g^−1^ DW) and aspartic acid (115.53 ± 4.85 mg g^−1^ DW) were the most abundant and represented (9.70 and 72.35%) of total AA contents of this algae. This composition quantified was more than the quantified amounts in the *C. racemosa* from Chinese waters with 15.27% and 3.63%, respectively [36]. According to several studies available, glutamic acids and aspartic acids constitute the main fraction of the total amino acid in the genus *Caulerpa* [38]. The abundant presence of these amino acids in the cultivated *C. racemosa* suggest that the alga has a potential value of flavor enhancers and tasty food [50,57]. The amino acid (AAs) composition of proteins in cultivated *C. racemosa* is illustrated in Table 3.

### 3.4. Mineral Contents

Seaweed is widely consumed for its natural vitamins, minerals, and plant-based protein. However, there has been great concern about heavy metals’ bioaccumulation from anthropogenic sources such as mining, petrochemical industry, printing, electronic industry, and municipal waste. The cell wall chemistry of the marine macroalgae comprises anionic carboxyl, sulfate, and phosphate groups responsible for the complexation of metallic cation. Their ability to bioaccumulate these elements depends on the presence of mineral in the environment, growth medium, sampling site, and the uptake capacity of the algae [58]. The cultivated *C. racemosa* samples contained significant amounts of essential minerals. The current analysis quantifies the samples with 26.61 ppm of calcium, followed by sodium (0.081 ppm DW), potassium (0.055 ppm DW) and magnesium (0.032 ppm DW). The ratio of Na/K (1.47) being relatively low is advantageous since high Na/K ratio diets are often related to the incidence of hypertension [39]. The ratio calculated in this sample was comparatively lower than several other reports within the genus Caulerpa [14,50]. This characteristic makes the cultivated *C. racemosa* would be a suitable substitute for medical patients who take diuretics to control hypertension and suffer from excessive secretion of potassium. Seaweeds with low ratios of Na/K are ideal for sodium chloride replacement [59]. Calcium-rich cultivated *C. racemosa* suggests that its intake could help to prevent a variety of bone-related diseases such as osteoporosis that are essential in intracellular functions and blood clotting hemostasis [60]. The analysis of microelements revealed that cultivated *C. racemosa* contained a notable amount of iron quantified at 23.74 ppm, followed by Mn (7.33 ppm DW) and zinc (0.032 ppm DW). Intake of iron is important for the body mainly during growth, pregnancy and deficiency would lead to anemia [61]. The presence of toxic metals such as arsenic (As), cadminum (Cd), lead (Pb) and aluminium (Al) were all found to be below the toxic limits. Therefore, the cultivated *C. racemosa* may be used as a food supplement to provide the daily intake of minerals for humans. The mineral composition of the cultivated *C. racemosa* is shown in Table 4.

### 3.5. Anti-Oxidative Properties

#### 3.5.1. Total Phenol Content (TPC) and Total Flavonoid Content (TFC).

Methanolic extraction of *C. racemosa* samples yielded an average of 26.6% crude extract of the total biomass. As a solvent of aqueous polarity, methanol was used to extract the seaweed collected from fish ponds in order to optimize the extraction of phenolics that contributes an essential role as an antioxidant agent [62,63]. The antioxidative potential of seaweed extracts are well exhibited and different extraction solvents are known to contribute to varying antioxidant properties [16,64]. The consumption of seaweed as an antioxidant rich food increased due to their protective effects against reactive oxygen species (ROS) which induced oxidative damage in human cells leading to various chronic diseases. The presence of natural antioxidants such as phenolics and flavonoids that bear hydroxyls functional groups in seaweed, which, among other nutritional properties, makes it an important functional food to inhibit oxidative stress donating hydrogen to stabilize and prevent the generation of free radicals [7,16,65], enabling it to reduce disease risk and health promotion [66]. The TPC and TFC content in the analysed seaweed samples were quantified as 17.88 ± 0.78 mg GAE g^−1^ and 59.43 ± 2.45 mg QE g^−1^, respectively. A recent report on wild Malaysian *C. racemosa* of identical extraction technique quantified the TPC and TFC content as 10.33 ± 0.02 mg GAE g^−1^ and 24.52 ± 2.17 mg QE g^−1^. It also exhibited the differences in choice of solvent and though the use of chloroform displayed the highest TPC level, and TFC content was second highest compared to water. This study suggested that the samples studied contained mostly less polar phenolics [16]. Flavonoids are a subgroup of phenolic compounds; therefore, these components are dependent on each other. Phenolics have the ability to scavenge free radicals, inhibit lipid peroxidation and upregulate certain metal-chelating reactions [65,67]. Abiotic factors such as levels of irradiance levels, temperature, nutrient availability, precipitation, salinity [47] and biotic factors such as grazing pressure, reproductive state and stage of life cycle affect polyphenols content in seaweed. As the seaweed analysed were collected from earthen fish ponds which is high in nutrients, this factor could contribute to levels of phenolic and flavonoid in the seaweed, affecting its antioxidant capacity [66].

#### 3.5.2. Antioxidant Capacity

Natural antioxidants such as flavonoids and phenolic acids have demonstrated a positive effect on human health as electron donors, either as reducing agents or inhibitors of peroxyl radicals to prevent cell damage by reactive oxygen species (ROS). The antioxidant effectiveness in a biological system is measured by the inhibition of oxidation through lipid peroxidation or free radical scavenging ability through varying mechanisms of actions [68]. Here, we evaluated the antioxidant capacity of the *C. racemosa* extracts through the total antioxidant capacity (TAC), ferric reducing antioxidant power (FRAP), and hydrogen peroxide radical scavenging capacity (H_2_O_2_) assays. The total antioxidant capacity of *C. racemosa* extracts was quantified as 0.31 ± 1.84 mg AAE g^−1^ extract. In a separate evaluation, the TAC of the Indian *C. racemosa* population methanolic extracts was quantified at 0.74 ± 0.13 mg AAE g^−1^ extract, which is twice as high as the current value. Several other species within the genus *Caulerpa* was also evaluated for its antioxidative capacity where *C. veravalnensis* and *C. scalpeliformis* recorded values of 0.46 ± 0.05 mg AAE g^−1^ and 0.42 ± 0.07 mg AAE g^−1^ extract, respectively. The ferric reducing capacity of *C. racemosa* extracts was quantified at 6.24 ± 3.03 μg AAE mL^−1^. The FRAP assay measures the capacity of an antioxidant compound to reduce ferric oxidant (Fe^3+^) to a ferrous complex (Fe^2+^) in a redox-linked colorimetric reaction by electron transfer, which specifies the capacity of the compound to reduce reactive species [69]. The H_2_O_2_ scavenging activity cultivated *C. racemosa* extracts were recorded at an average of 80.55 ± 0.55%. Several studies have also reported the potentially high H_2_O_2_ scavenging properties of seaweeds [28]. The antioxidative properties of the cultured *C. racemosa* samples are displayed in Table 5.

A correlation analysis was done to evaluate the relationship between TPC, TFC and antioxidant capacity of the investigated *C. racemosa* samples. The phenolics in cultivated *C. racemosa* exhibited a weak positive correlation of ferric reduction (r = 0.6612; R^2^ = 0.4372) and H_2_O_2_ scavenging potential (r = 0.3775; R^2^ = 0.1425). Conversely, there was a negative correlation between phenolics and total antioxidative capacity (r = −0.9039; R^2^ = 0.8169). Previous studies have shown a positive correlation between TPC and FRAP in *C. racemosa* [7,70]. The reducing property of the extracts are strongly related to the presence of reductants that are compounds responsible for the ferric reduction potentials in this species [65]. The flavonoid content of cultivated *C. racemosa* displayed strong correlation with the total antioxidant capacity (r = 0.9957; R^2^ = 0.991), but a weak correlation with H_2_O_2_ (r = 0.4616; R^2^ = 0.2131). A poor positive correlation was observed between ferric reduction capacity and flavonoids content in the collected samples (r= −0.6162; R^2^ = 0.3797). This is in line with other studies on the methanolic extract of *Caulerpa* spp that have reported a positive correlation for both the TFC and H_2_O_2_ [7,68]. The positive correlation between phenolics, flavonoids and antioxidant capacity values as recorded by the various assays performed is affected by the nature of metabolites present in the seaweed such as bisindole alkaloids, terpenoids, protein or peptides and chlorophyll pigments [13,70]. The antioxidative properties of the investigated seaweed samples are summarized in Table 6.

### 3.6. In Vivo Anti-Diabetic Activity

Unlike other diseases, screening natural extracts and drug molecules for antidiabetic bioactivity are essentially conducted in animal models. This is because the processes and mechanisms leading to diabetes and its complications involve more than one organ. In vitro results are seldom translated into meaningful in vivo outcomes, especially in a disease such as diabetes mellitus, as only a specific cell line or a specific organ is normally targeted in in vitro studies. In vivo studies on specialized animal models have allowed great progress in tailoring research questions towards individualized genetic and biochemical contributors and their effect on the pathogenesis of the disease processes [71].

In this study, thirty SD rats were divided into five groups of six rats each. The groupings are displayed in Table 1. Group I was the control. All overnight (12 h) fasted rats in the groups Group II to Group V was induced with streptozotocin (STZ) via intraperitoneal injection. The hyperglycaemia level of the rats were confirmed by measuring the blood glucose level of diabetic rats 10 days post-streptozotocin injection by the tail–vein method. Animals that showed blood glucose levels higher than 11.1 mmol L^−1^ were considered diabetic. All confirmed diabetic rats were administered with the ethyl acetate extract of pond cultured *C. racemosa*. Two doses, low (100 mg kg^−1^) and high (200 mg kg^−1^), were administered and compared to metformin as standard. The glucose level of the experimental rats as monitored during the 14-day treatment period. Throughout the 14-day treatment, body weight and food intake were monitored. At the end of the 14-day treatment, plasma, albumin and cholesterol levels were quantified and histopathology of pancreas, liver and kidney was performed to observe for irregularities.

#### 3.6.1. Effect of *C. racemosa* Etoac Extract on the Body Weight and Food Intake

**Figure 1:**
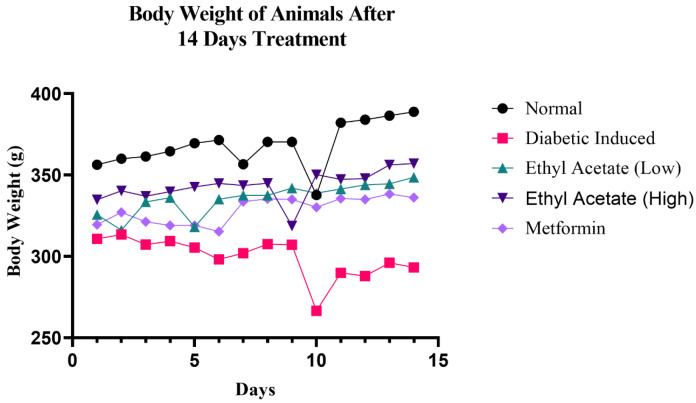
Changes in body weight in diabetic induced rat after 14 days of cultivated *C. racemosa* EtOAc extract treatment.

Body weight of the test animals was assessed throughout the 14 days of treatment and the results are shown in Figure 1. It can be observed that the rats in the diabetic-induced group (Group II) exhibited constant weight loss throughout the 14-day treatment. However, the greatest dip was observed on day 9 of treatment, possibly due to the deteriorating insulin level, unlike the control where random reduction was observed; as a whole, there was a positive trend throughout the observation. The two groups that received low (100 mg kg^−1^) and high (200 mg kg^−1^) doses of cultivated *C. racemosa* extract exhibited reduction of body weight in days 5 and 9, respectively, but managed to recover the increment of body weight towards the end of the 14-day treatment. Therefore, it is evident than the *C. racemosa* EtOAc extract was effective in preventing weight loss in diabetic induced rats.

**Figure 2:**
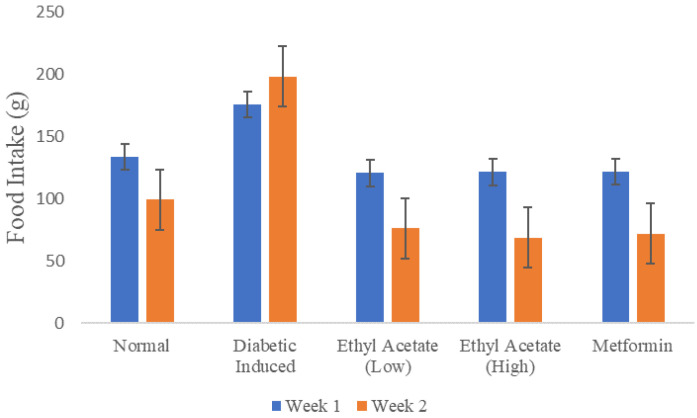
Food intake of animals during the 14 days of treatment.

Figure 2 shows the recorded food intake of the animals in throughout the 14-day treatment monitored on a weekly basis. The total amount of food consumed by the diabetic-induced group during the second week of treatment was more than food consumed in the first week compared to other groups. As a comparison, the EtOAc treated rats and both positive, negative control exhibited at least a consistent 25% reduction food intake in week 2.

Although the diabetic-induced group consumed more food compared to other groups, it had a significantly lower body weight comparatively. This could be attributed to the ineffective glucose utilization which results in the protein and fat metabolism. Body weight is positively influenced by structural proteins. Thus, the breakdown of these proteins leads to a decrease in body weight. Weight loss is observed during relative or absolute insulin deficiency due to the production of adenosine triphosphate (ATP) and decrease in the synthesis of protein in all tissues [72]. In this experiment, the *C. racemosa* ethyl acetate extract has a weight-loss preventing, nutritive effect in diabetic induced rats.

#### 3.6.2. Effects of *C. racemosa* Etoac Extract on Plasma Glucose Levels

**Figure 3:**
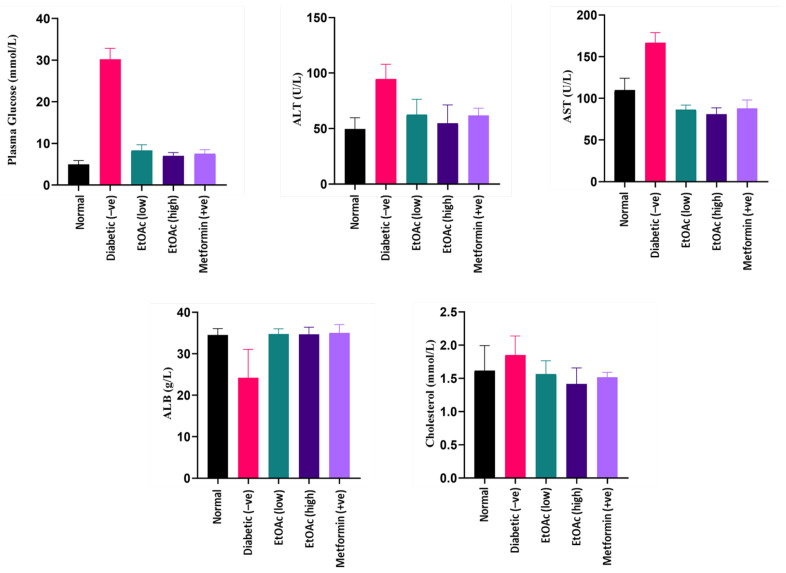
Plasma glucose, ALT, AST, albumin and cholesterol levels of diabetic rats treated with cultivated *C. racemosa* EtOAc extracts.

Blood plasma obtained after 14 days of treatment was used to estimate the plasma glucose levels of the animals. Due to the diabetic induction, rats in group II were observed to contain the highest plasma glucose level. After 14 days, a significant reduction in the plasma glucose level of the animals was observed when the diabetic rats were treated with high (200 mg kg^−1^) and low (100 mg kg^−1^) doses of *C. racemosa* extract. Based on the plasma analysis, a *C. racemosa* treated group had shown a significant decrease (*p* < 0.05) in blood glucose levels as compared to the untreated diabetic group. The *C. racemosa* extract-treated group demonstrated similar efficacy in lowering the blood glucose as Metformin. Hence, the anti-hyperglycaemic effect of *C. racemosa* extract is evident. A hyperglycaemic state may induce oxidative stress that could be detrimental to insulin-sensitive tissues such as liver which may cause damage to the organ [73,74,75]. Plasma glucose levels of the cultivated *C. racemosa* EtOAc extracts are shown in Figure 3.

#### 3.6.3. Effect of *C. racemosa* Ethyl Acetate Extract on Plasma ALT and AST Levels

Liver function tests (LFTs) such as AST and ALT were analysed as their values reflect the liver function in both normal and diabetic animals. These two LFTs serve as biomarkers for liver damage as they measure the intracellular liver enzyme concentration that have leaked into blood circulation, which is a classic characteristic of hepatocyte injury [76]. According to the results, both AST and ALT readings of *C. racemosa* extract-treated groups (Group III and IV) demonstrated significantly lower levels (*p* < 0.05) compared to the STZ-induced diabetic group (Group II). Hence, it can be proposed that *C. racemosa* extract may have a hepatoprotective effect in diabetic-induced rats.

The diabetic-induced group had significantly high ALT levels as compared to the other groups. The treatment of *C. racemosa* EtOAc extract consistently exhibited a decrease in ALT levels in the diabetic induced rats. Thus, a *C. racemosa* extract had the potential to reduce the elevated ALT levels in diabetic animals. Likewise, diabetic rats that received *C. racemosa* extract treatment showed a significantly lower AST levels compared to the normal and untreated diabetic-induced groups. The AST levels of the diabetic-induced rats naturally contain high AST levels. Both of these observations indicate that doses of *C. racemosa* EtOAc extract up to 200 mg kg^−1^ concentration are not lethal and have the potential to maintain the level of ALT and AST under diabetic condition. ALT and AST levels of the cultivated *C. racemosa* EtOAc extracts are shown in Figure 3.

#### 3.6.4. Effects of *C. racemosa* Etoac Extract on Albumin Levels

Persistent microalbuminuria is a classic clinical manifestation of diabetic nephropathy. It occurs commonly in Type 1 diabetes. A persistently elevated blood sugar level may cause kidney injury directly or via hemodynamic modifications. It does so by inducing the protein kinase C activation, increasing the advanced glycosylation end (AGE) products formation and the synthesis of diacylglycerol. Eventually, these will lead to the occurrence of sheer stress, glomerular hyperfiltration and microalbuminuria [76]. Improper functioning of the kidney will cause the albumin to leak into the urine, which explains the lower serum albumin level in diabetic nephropathy patients. In this study, rats treated with doses of *C. racemosa* EtOAc extracts exhibited a significantly higher plasma albumin level (ABL) (*p* < 0.05) in comparison with the diabetic rats (Group II), which had the lowest albumin levels among all groups. Both low (100 mg kg^−1^) and high (200 mg kg^−1^) doses of cultivated *C. racemosa* extract-treated groups plasma albumin levels similar to normal and Metformin-treated rat groups, indicating pharmacological possibilities in the seaweed extract as a nephroprotective agent. Plasma albumin levels of the cultivated *C. racemosa* EtOAc extracts are shown in Figure 3.

#### 3.6.5. Effects of *C. racemosa* Etoac Extract on Cholesterol Levels

Insulin plays a role as a key hormone in regulating the metabolism of lipid, being an anti-lipolytic, insulin inhibit hormone-sensitive lipase in adipose tissue. It decreases the circulating non-esterified fatty acids (NEFA) secretion and promotes triacylglycerols storage in adipocytes [77]. Deficiency of insulin in diabetes could affect lipid metabolism, which in turn may be manifested as a high cholesterol reading. According to the results from the study, cholesterol readings between all groups fall in a normal range without any significant differences. It can be deduced that the *C. racemosa* EtOAc extract has little to no effect on the cholesterol level of diabetic-induced rats, which further adds to the nutritive value of the species. Cholesterol levels of the cultivated *C. racemosa* EtOAc extracts are shown in Figure 3.

## 4. Conclusions

The cultivated *C. racemosa* obtained from culture ponds at Johor contains the necessary composition of carbohydrate, protein, calories, ash, low lipid content combined with essential amino acids, mineral contents, and polyunsaturated acids, making it a potential candidate as an alternative source of functional food. The EtOAc extracts of *C. racemosa* exhibited promising antioxidant, reducing and antidiabetic activities. As *C. racemosa* is cultivated in small-scale farming in Malaysian waters, this study supports the potential of cultured *C. racemosa* to serve as functional food with therapeutic applications beneficial to human health. Future studies can consider investigating the specific bioactive constituents that are responsible for the therapeutic activities exhibited.

## Figures and Tables

**Table 1:** Animals groupings used in assessing the antihyperglycaemic potentials of *C. racemosa* extracts.

Grouping	Characteristics
Group I	Normal control group
Group II	Diabetes-induced group
Group III	*C. racemosa* EtOAc extract treated group (100 mg kg^−1^ body weight)
Group IV	*C. racemosa* EtOAc extract treated group (200 mg kg^−1^ body weight)
Group V	Standard Metformin treated group

**Table 2:** The proximate composition of cultivated *C. racemosa* (%, *w/w* on the dry basis).

Component	Pond 1	Pond 2	Pond 3	Average
**Proximate Composition**
Moisture	13.807	14.807	12.94	13.85 ± 0.93
Ash	28.558	28.097	28.097	28.25 ± 0.27
Crude lipid	4.092	4.562	3.949	4.20 ± 0.32
Protein	20.15	20.43	20.24	20.27 ± 0.14
Carbohydrate	33.393	32.104	34.774	33.42 ± 1.34
Caloric Value (cal g^−1^)	2547.45	2549.9	2536.66	2544.67 ± 7.04
Vitamin B2 (mg kg^−1^)	2.00	2.00	2.10	2.03 ± 0.06

Each value in the table is represented as mean ± SD (*n* = 3).

**Table 3:** Gas chromatography determination of fatty acid composition (g fatty acid methyl esters/100 g total fat) of cultivated *C. racemosa*.

Fatty Acids Profile	Pond 1	Pond 2	Pond 3	Average
**SFA**
Lauric acids (C12:0)	2.50	2.40	2.50	2.47 ± 0.06
Myristic acid (C14:0)	4.40	4.30	4.30	4.33 ± 0.06
Palmitic acid (C16:0)	63.50	63.10	63.20	63.27 ± 0.21
Stearic acid (C18: 0)	4.70	4.80	4.70	4.73 ± 0.06
Behenic acid (C22:0)	1.40	1.40	1.40	1.40 ± 0.00
Lignoseric acid DHA (C24:0)	4.30	4.50	4.30	4.37 ± 0.12
Total				80.57 ± 0.12
**MUFA**
Palmitoleic acid (C16:1)	3.90	3.80	3.90	3.87 ± 0.06
Oleic acid(C18:1)	5.70	5.80	5.90	5.80 ± 0.10
Erucic acid (C22:1)	1.10	1.20	1.10	1.13 ± 0.06
Nervonic acid (C24:1)	1.40	1.50	1.40	1.43 ± 0.06
Total				12.23 ± 0.07
**PUFA**
α-Linolenic acid (C18:3)	1.80	1.80	1.90	1.83 ± 0.06
Linoleic acid (C18:2)	5.30	5.30	5.40	5.33 ± 0.06
Total				7.16 ± 0.07
ω6/ω3				2.91 ± 0.03

Each value in the table is represented as mean ± SD (*n* = 3). Saturated fatty acids: SFA. Monounsaturated fatty acids: MUFA. Polyunsaturated fatty acids: PUFA.

**Table 4:** Amino acid content in cultivated *C. racemosa* (mg g^−1^ protein).

Amino Acids	Pond 1	Pond 2	Pond 3	Average
**Essential Amino acids**
Threonine (Thr)	1.49	1.39	1.52	1.42 ± 0.60
Valine (Val)	1.45	1.37	1.45	1.42 ± 0.49
Phe + Tyr (TAA)	1.21	1.29	1.22	1.24 ± 0. 43
Isoleucine (Ile)	0.83	0.82	0.81	0.82 ± 0.11
Leucine (Leu)	2.21	2.43	2.39	2.34 ± 1.16
Lysine (Lys)	0.40	0.43	0.44	0.42 ± 0.21
Met + Cys (SAA)	ND	ND	ND	ND
Histidine	ND	ND	ND	ND
Tryptophan (Try)	ND	ND	ND	ND
∑ EAAs	7.59	7.73	7.67	7.68 ± 0.81
**Non-essential acids**
Aspartic acid (Asp)	115.73	115.88	114.97	115.53 ± 4.85
Glutamic acid (Glu)	15.94	15.31	15.23	15.49 ± 3.87
Serine (Ser)	1.52	1.54	1.58	1.55 ± 0.27
Glycine (Gly)	1.07	1.13	1.21	1.14 ± 0.69
Arginine (Arg)	3.06	3.01	3.08	3.05 ± 0.38
Alanine (Ala)	0.78	0.79	0.77	0.78 ± 0.08
Tyrosine (Tyr)	6.51	6.53	6.51	6.52 ± 0.10
Cysteine (Cys)	2.54	2.74	2.57	2.62 ± 1.06
Proline (Pro)	5.47	5.34	5.23	5.35 ± 1.20
Asparagine (Asn)	ND	ND	ND	ND
Glutamine (Gln)	ND	ND	ND	ND
∑ NEAA	152.62	152.27	151.15	152.02 ± 8.22
∑ AA	160.21	160.00	158.82	159.69 ± 7.41
∑ EAAs/∑ NEAAs (ratio)				0.05

Values are mean ± SD (*n* = 3). Not detected (ND); Amino acids (AAs); Essential amino acids (EAAs); Non-essential amino acids (NEAAs).

**Table 5:** Mineral content in cultivated *C. racemosa*.

Minerals	Pond 1	Pond 2	Pond 3	Average
**Macro Mineral (ppm)**
Calcium (Ca)	26.30	26.12	27.41	26.61 ± 0.70
Magnesium (Mg)	0.040	0.036	0.020	0.032 ± 0.01
Potassium (K)	0.055	0.054	0.056	0.055 ± 0.001
Sodium (Na)	0.078	0.089	0.076	0.081 ± 0.007
**Trace Mineral (ppm)**
Copper (Cu)	0.008	0.012	0.010	0.010 ± 0.002
Iron (Fe)	22.98	24.38	23.86	23.74 ± 0.71
Manganese (Mn)	7.48	7.26	7.25	7.33 ± 0.13
Selenium (Se)	0.004	0.005	0.003	0.004 ± 0.001
Zinc (Zn)	0.048	0.024	0.024	0.032 ± 0.014
Chromium (Cr)	0.002	0.004	0.003	0.003 ± 0.001
Cobalt (Co)	0.002	0.002	0.002	0.002 ± 0.00
**Heavy metal (ppb )**
Total Arsenic (As)	3.00	3.00	3.00	3.00 ± 0.00
Cadmium (Cd)	-	-	-	<MDL
Lead (Pb)	4.52	4.84	4.65	4.67 ± 0.16
Aluminium (Al)	1798.33	1890.67	1967.95	1885.65 ± 84.92

DW (Dry Weight); MDL (Method detection limit); Triplicate measurements (mean (SD), n = 3) of each sample with RSD is less than 10%; ppb = Parts per billion; ppm = Parts per million.

**Table 6:** Antioxidative properties of *C. racemosa* and its correlation with phenolics and flavonoids content.

*C. racemosa*	Total Phenolic Content	Total Flavonoid	Extraction Yield
	(mg GAE g^−1^)	(mg QE g^−1^)	(%)
Pond 1	17.23 ± 0.55	58.90 ± 3.22	31.8
Pond 2	17.52 ± 1.78	59.52 ± 1.58	25.7
Pond 3	18.89 ± 0.82	59.87 ± 2.55	22.3
Average	17.88 ± 0.78	59.43 ± 2.45	26.6
		**Correlation (R^2^)**	
	**Radical Scavenging Value**	**Phenols**	**Flavonoids**
**TAC**			
Pond 1	0.26 ± 1.45
Pond 2	0.98 ± 2.13	0.8169	0.3797
Pond 3	0.48 ± 1.94		
Average	0.31 ± 1.84		
**FRAP**			
Pond 1	6.05 ± 1.98
Pond 2	5.79 ± 2.55	0.4372	0.991
Pond 3	6.88 ± 4.56
Average	6.24 ± 3.03		
**H_2_O_2_**			
Pond 1	80.24 ± 0.23
Pond 2	80.63 ± 0.84	0.1425	0.2131
Pond 3	80.78 ± 0.58
Average	80.55 ± 0.55		

Each value in the table is represented as mean ± SD (*n* = 3).
